# Molecule Non-Radiative Coupling to a Metallic Nanosphere: An Optical Theorem Treatment

**DOI:** 10.3390/ijms10093931

**Published:** 2009-09-08

**Authors:** Gérard Colas des Francs

**Affiliations:** Institut Carnot de Bourgogne, UMR 5209 CNRS - Université de Bourgogne, 9 Av. A. Savary, BP 47 870, 21078 Dijon, France; E-Mail:gerard.colas-des-francs@u-bourgogne.fr

**Keywords:** surface enhanced spectroscopy, energy transfer, optical theorem, plasmon modes

## Abstract

The non-radiative coupling of a molecule to a metallic spherical particle is approximated by a sum involving particle quasistatic polarizabilities. We demonstrate that energy transfer from molecule to particle satisfies the optical theorem if size effects corrections are properly introduced into the quasistatic polarizabilities. We hope that this simplified model gives valuable information on the coupling mechanism between molecule and metallic nanos-tructures available for, *e.g.,* surface enhanced spectroscopy signal analysis.

## 1. Introduction

The interaction between molecules and metallic particles has been extensively studied since the pioneer work of Gersten and Nitzan in the 1980s [1]. Indeed, surface enhanced spectroscopies techniques profit from the strong excitation and emission rates enhancement for molecules located in close proximity of metallic surface rugosities. In particular, giant Raman signals have been recorded by surface enhanced Raman scattering (SERS) leading to chemical- and bio-sensing applications [2]. Although the electromagnetic coupling of a molecule to metallic nanostructures can be numerically investigated for arbitrary particles shapes, it is also fruitful to use some simplified model to deeply understand the underlying photophysical process. Recently, Polman and coworkers theoretically investigated the coupling of molecules to spheroidal particles [3, 4]. To this aim, they significantly improved the quasistatic model previously developed by Gersten and Nitzan. Treating retardation effects as a perturbation to the quasi-static case, they were able to precisely point out the particles modes involved in the coupling process. However, they solely consider correction to the dipolar mode whereas higher orders modes play an important role, particularly in the non-radiative contribution. In this communication, we extend the correction factor to all the particles modes. Specifically, we discuss the energy conservation, in connection with the optical theorem, for the non-radiative coupling rate. This discussion carries on our recent descriptions of energy transfer in near field optics [5–7].

## 2. Non-Radiative Coupling Rate in the Quasi-Static Regim

### 2.1. Expansion on Particles Modes Polarizabilities

**Figure 1.**
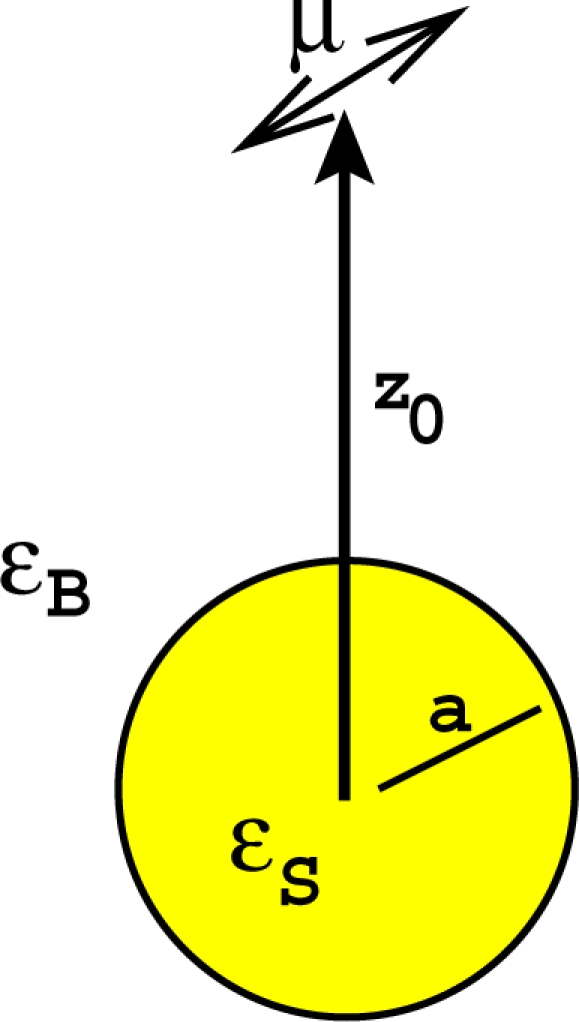
Molecule-particle geometry. The molecule dipole moment *μ* is located at a distance *z*_0_ from the particle center.

The electromagnetic coupling of a single molecule to a metallic particle is classically described as the interaction of the molecule dipole transition moment with a sphere of radius *a* and complex dielectric constant *ε_S_* (Figure 1). The coupled system is embedded in an homogeneous background of real dielectric constant $n_{B}^{2}$. The molecule-particle center separation distance is *z*_0_ and *k_B_* = *n_B_ω/c* is the wavevector in the homogeneous medium at the molecule emission frequency *ω*.

Using Mie formulation, we recently achieved an exact expression of the molecule normalized non-radiative decay rate, *γ^NR^/γ*_0_. This involves the Mie scattering coefficients *A_n_* and *B_n_* of the particle and spherical Hankel ( $h_{n}^{\left(1\right)}$) or Ricatti-Bessel ( $\zeta_{n}^{\left(1\right)}$) functions [5]

(1) $$\begin{array}{l} \frac{\gamma_{⊥}^{N\;R}}{\gamma_{0}}=\frac{3n_{B}}{2}\;\sum_{n=1}^{\infty }n(n+1)\;(2n+1)\;\;{\left|\frac{h_{n}^{\left(1\right)}(k_{B}\;z_{0})}{{\left(k_{B}\;z_{0}\right)}^{2}}\right|}^{2}\;\left[\text{Re}\left(-B_{n}\right)-{\left|B_{n}\right|}^{2}\right]\\ \frac{\gamma_{\left|\right|}^{N\;R}}{\gamma_{0}}=\frac{3n_{B}}{2}\;\sum_{n=1}^{\infty }(n+\frac{1}{2})\;\;[{\left|\frac{\zeta_{n}^{\left(1\right)}(k_{B}\;z_{0})}{{\left(k_{B}\;z_{0}\right)}^{2}}\right|}^{2}\;\;\left[\text{Re}\;(-B_{n})-{\left|B_{n}\right|}^{2}\right]+|h_{n}^{\left(1\right)}\;(k_{B}\;z_{0})\;{|}^{2}[\text{Re}\;(-A_{n})-|A_{n}{|}^{2}]] \end{array}$$

where subscripts (⊥) and (‖) denote a molecule perpendicular or parallel to the particle surface, respectively. In these exact expressions, terms proportional to *Re*(*−C_n_*) (*C_n_* = *A_n_, B_n_*), represent the extinction of the light emitted by the excited molecule, through the *n^th^* particle mode channel. Terms proportional to *|C_n_|*^2^ correspond to the scattering emitted light of the coupled molecule–particle system, also through the *n^th^* particle mode channel. Since extinction is the sum of dissipation into the particle and scattering in the far field, the energy transfer rate from molecule to metallic particle is given by the difference between extinction and scattering rates, on all the particles modes, in agreement with the optical theorem.

We are now interested in the optical theorem verification when the quasistatic form of the decay rates are considered instead of the exact Mie expansion given above. We start from the approximated non-radiative rates, deduced from expressions (1,2) by taking the quasistatic limit *k_B_z*_0_ *→* 0 [5]

(2) $$\begin{array}{l} \frac{\gamma_{⊥}^{N\;R}}{\gamma_{0}}\approx \frac{3}{2}n_{B}\frac{1}{{\left(k_{B}\;z_{0}\right)}^{3}}\;\sum_{n=1}^{\infty }\frac{{\left(n+1\right)}^{2}}{z_{0}^{\left(2n+1\right)}}\\ \;\;\;\;\;\;\;\;\;\;\;\;\;\;\;\;\;\left[Im(\alpha_{n})-\frac{(n+1)\;k_{B}^{2n+1}}{n(2n-1)!!(2n+1)!!}|\alpha_{n}{|}^{2}\right]\;\text{and} \end{array}$$

(3) $$\begin{array}{l} \frac{\gamma_{\left|\right|}^{N\;R}}{\gamma_{0}}\approx \frac{3}{4}n_{B}\frac{1}{{\left(k_{B}\;z_{0}\right)}^{3}}\;\sum_{n=1}^{\infty }\frac{n(n+1)}{z_{0}^{\left(2n+1\right)}}\\ \;\;\;\;\;\;\;\;\;\;\;\;\;\;\;\;\;\left[Im(\alpha_{n})-\frac{(n+1)k_{B}^{2n+1}}{n(2n-1)!!(2n+1)!!}|\alpha_{n}{|}^{2}\right] \end{array}$$

where we use the notation (2*n* − 1)!! = 1 × 3 × . . . × (2*n* − 1) and *α_n_* is the *n^th^* order multipolar polarizability of the sphere

(4) $$\alpha_{n}=\frac{n({ɛ}_{S}-{ɛ}_{B})}{(n+1)\;{ɛ}_{B}+n{ɛ}_{S}}a^{\left(2n+1\right)}$$

Resonances occur at frequencies *ω_n_* such that ${ɛ}_{S}=-\frac{\left(n+1\right)}{n}{ɛ}_{B}$. This corresponds to negative *ε_S_*, *i.e.,* metallic behaviour. Actually, at the resonance, the metal free electrons coherently oscillate, forming a “plasma quasi-particle”. These are the so-called plasmon modes. Silver and gold nanoparticles support plasmon modes in the visible range so that they efficiently couple to fluorescent molecules. Obviously, depending on the molecule emission frequency, different plasmon modes would be involved in the coupling process.

Physically, when the metallic particle is illuminated with a non-uniform electric field wave **E**(**r**), a series of plasmon modes can be excited, namely, the dipolar (n = 1), quadrupolar (n = 2), . . ., 2*^n^*-polar modes. The *n^th^* multipole tensor moment is given by [8]

(5) $${\text{p}}^{\left(n\right)}=\frac{4\pi \;{ɛ}_{0}{ɛ}_{B}}{(2n-1)!!}\alpha_{n}\;\nabla^{n-1}\text{E}$$

For instance, only the dipole moment **p**^(1)^ = 4*πε*_0_*ε_B_α*_1_**E** exists in an uniform field. On the contrary, fast spatial variations of the electric field emitted in the near field of a molecule lead to strong field gradients so that high orders modes can be excited as seen from the sum involved in Equation 2 or 3.

### 2.2. Optical Theorem and Finite Size Corrections

**Figure 2.**
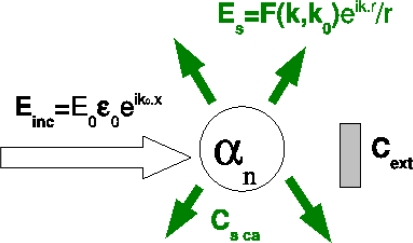
Multipolar mode cross-sections definition and optical theorem. An incident plane wave *E_inc_* excites the particule, characterized by its *n^th^* order multipolar polarizability *α_n_*. The extinction cross-section *C_ext_* is the ratio of the power taken from the incident wave to the incident power per unit area. The optical theorem connects the extinction cross-section to the imaginary part forward scattering amplitude, namely, *C_ext_* = 4*π/*(*kE*_0_)*Im*[***ε*_0_*·* F⋆(k = k_0_**] [10]. The scattering cross-section corresponds to the intensity scattered in the whole far-field space. In case of absorbing material, an additionnal dissipative channel is the absorption within the particle.

Nevertheless, Equations 2 and 3 lead to a wrong result when applied to a non-dissipative particle (*ε_S_* real). Indeed, then *Im*(*α_n_*) = 0 for all *n* and the non-radiative coupling rate is negative. This originates from the neglected retardation effects. This apparent problem is well-known from the application of the optical theorem to a dipolar transparent sphere [9]. Indeed, let us consider the extinction cross-section *C_ext_* of a small dielectric particle. *C_ext_* is the sum of absorption (*C_abs_*) and scattering (*C_sca_*) cross-sections; *C_ext_* = *C_abs_* + *C_sca_* (Figure 2). For a dipolar particle, the scattering cross-section is given by $C_{\text{sca}}=(8\pi /3)\;k_{B}^{4}|\alpha_{1}{|}^{2}$. In addition, applying the optical theorem, the extinction cross-section expresses *C_ext_* = 4*πk_B_Im*(*α*_1_). This last relation is no more valuable for a non absorbing particle (*C_abs_* = 0 and *Im*(*α*_1_) = 0) since then *C_ext_* = 0 ≠ *C_sca_*.

This can be overcome taking into account the particle finite size. An exact expression of the particle dipolar polarizability can be obtained for regular shapes [11]. However, an approximated expression is easily deduced from the optical theorem. Indeed, *C_ext_* = *C_abs_* + *C_sca_* is verified, even for a non-absorbing sphere, if we replace the quasi-static dipolar polarizability *α*_1_ by an effective polarizability $\alpha_{1}^{e\;f\;f}={\left[1-i(2/3)\;k_{B}^{3}\alpha_{1}\right]}^{-1}\alpha_{1}$. The corrective term ( $-i2/3\;k_{B}^{3}\alpha_{1}$) is called the radiative reaction correction since it microscopically originates from the radiation emitted by the moving charges that has to be self-consistently introduced into the charges motion Equation [10].

A close inspection of relations (2) and (3) suggests to define similarly effective *n^th^* order multipolar polarizabilities

(6) $$\alpha_{n}^{e\;f\;f}={\left[1-i\frac{(n+1)k_{B}^{2n+1}}{n(2n-1)!!(2n+1)!!}\alpha_{n}\right]}^{-1}\;\alpha_{n}$$

The non-radiative coupling expressions are now modified, by analogy with the dipolar case [12], to

(7) $$\begin{array}{l} \frac{\gamma_{⊥}^{N\;R}}{\gamma_{0}}\approx \frac{3}{2}n_{B}\frac{1}{{\left(k_{B}\;z_{0}\right)}^{3}}\;\sum_{n=1}^{\infty }\frac{{\left(n+1\right)}^{2}}{z_{0}^{\left(2n+1\right)}}\\ \;\;\;\;\;\;\;\;\;\;\;\;\;\;\;\;\;\left[Im(\alpha_{n}^{e\;f\;f})-\frac{(n+1)k_{B}^{2n+1}}{n(2n-1)!!(2n+1)!!}|\alpha_{n}^{e\;f\;f}{|}^{2}\right]\;\text{and} \end{array}$$

(8) $$\begin{array}{l} \frac{\gamma_{\left|\right|}^{N\;R}}{\gamma_{0}}\approx \frac{3}{4}n_{B}\frac{1}{{\left(k_{B}\;z_{0}\right)}^{3}}\;\sum_{n=1}^{\infty }\frac{n(n+1)}{z_{0}^{\left(2n+1\right)}}\\ \;\;\;\;\;\;\;\;\;\;\;\;\;\;\;\;\;\left[Im(\alpha_{n}^{e\;f\;f})-\frac{(n+1)k_{B}^{2n+1}}{n(2n-1)!!(2n+1)!!}|\alpha_{n}^{e\;f\;f}{|}^{2}\right] \end{array}$$

so that the non-radiative decay rate is exactly cancelled for non-absorbing particles.

**Figure 3.**
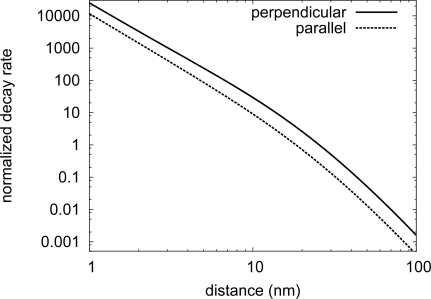
Non–radiative coupling in function of molecule-particle surface distance *d* = *z*_0_ *− a*. The gold particle radius is 15 nm and the molecule emission wavelength is 580 nm (correspondings to *e.g.,* terrylene molecule). The coupled system is immersed in water (*n_B_*=1.33). The log-log scale reveals the expected linear (*∝ d*^−3^) at very short distances.

Figure 3 presents the non-radiative coupling rates dependence on distance, using Equations 7 and 8. The fluorescence quenching as well as the quasi-static linear behaviour at short distances clearly appear [5].

Equations 7 and 8 extend expressions recently proposed by Mertens and Polman where finite size correction was applied to the dipolar polarizability only [3, 4]. Notably, they demonstrated that this correction strongly improve the numerical evaluation of the relaxation rates (both radiative and non-radiative contributions) of a molecule coupled to a metallic particle. They also consider molecule coupling to prolate particles for which no exact formulation exists, on the contrary to spherical particles. Importantly, the generalisation of the corrections to high-order modes proposed here also applies for prolate shapes. However, it is worthwhile to note that the dipolar correction is sufficient to numerically obtain a satisfactory agreement with exact calculations and almost no numerical improvement is achieved when extending the correction to high order modes. However it is a critical point to be considered in energy transfer description since the energy conservation is violated otherwise [6, 12, 13].

## 3. Conclusions

In conclusion, we obtain a simple expression for the non-radiative coupling rate of an excited molecule to a metallic spherical particle. The retardation effects are pertubatively introduced into the quasistatic polarizabilities. In this formulation, we achieve a consistent simplified model for which the energy conservation, or equivalently the optical theorem, is satisfied.
